# Clinical use of tigecycline may contribute to the widespread dissemination of carbapenem-resistant hypervirulent *Klebsiella pneumoniae* strains

**DOI:** 10.1080/22221751.2024.2306957

**Published:** 2024-01-19

**Authors:** Miaomiao Xie, Lianwei Ye, Kaichao Chen, Qi Xu, Chen Yang, Xiangnan Chen, Edward Wai-Chi Chan, Fuyong Li, Sheng Chen

**Affiliations:** a Department of Infectious Diseases and Public Health, Jockey Club College of Veterinary Medicine and Life Sciences, City University of Hong Kong, Kowloon, Hong Kong; b Department of Food Science and Nutrition, Faculty of Science, The Hong Kong Polytechnic University, Kowloon, Hong Kong; c State Key Lab of Chemical Biology and Drug Discovery, Department of Applied Biology and Chemical Technology, The Hong Kong Polytechnic University, Kowloon, Hong Kong

**Keywords:** Carbapenem-resistant hypervirulent *Klebsiella pneumoniae*, tigecycline, virulence plasmid, fitness, colonization

## Abstract

The emergence of carbapenem-resistant hypervirulent *Klebsiella pneumoniae* (CR-hvKP) poses grave threats to human health. These strains increased dramatically in clinical settings in China in the past few years but not in other parts of the world. Four isogenic *K. pneumoniae* strains, including classical *K. pneumoniae*, carbapenem-resistant *K. pneumoniae* (CRKP), hypervirulent *K. pneumoniae* (hvKP) and CR-hvKP, were created and subjected to phenotypic characterization, competition assays, mouse sepsis model and rat colonization tests to investigate the mechanisms underlying the widespread nature of CR-hvKP in China. Acquisition of virulence plasmid led to reduced fitness and abolishment of colonization in the gastrointestinal tract, which may explain why hvKP is not clinically prevalent after its emergence for a long time. However, tigecycline treatment facilitated the colonization of hvKP and CR-hvKP and reduced the population of *Lactobacillus* spp. in animal gut microbiome. Feeding with *Lactobacillus* spp. could significantly reduce the colonization of hvKP and CR-hvKP in the animal gastrointestinal tract. Our data implied that the clinical use of tigecycline to treat carbapenem-resistant *K. pneumoniae* infections facilitated the high spread of CR-hvKP in clinical settings in China and demonstrated that *Lactobacillus* spp*.* was a potential candidate for anticolonization strategy against CR-hvKP.

## Introduction

The development of carbapenem resistance and hypervirulence has been regarded as two different evolutionary directions of *Klebsiella pneumoniae* that led to the emergence of epidemic and high-risk strains. Carbapenem-resistant *K. pneumoniae* (CRKP) strains have posed an imminent challenge to the clinical treatment of *K. pneumoniae* infections due to the extended phenotypic drug resistance and high transmissibility of such strains, which are known to cause infections of much higher mortality than their carbapenem-susceptible counterparts [1]. Hypervirulent *K. pneumoniae* (hvKP) has also emerged as a superbug that causes severe pyogenic liver abscesses and aggressive infections [2]. *K. pneumoniae* isolates that encode both phenotypes, namely carbapenem-resistant hypervirulent *K. pneumoniae* (CR-hvKP), which can cause infections that are even more fatal and harder to treat when compared to infections caused by CRKP and hvKP, have emerged [3–5]. Recent evidence showed that these newly emerged CR-hvKP strains start to cause severe invasive infections of high mortality worldwide, even among healthy individuals [2]. In recent years, CR-hvKP in particular ST11 type has been widely reported in clinical settings in China [6]. A recent report on surveillance of CRKP in various countries showed that CRKP not only exhibited a high prevalence rate of 27% in China, but a large proportion of such strains (69%) also harboured plasmid-related virulence genes such as *rmpA/A2*, *iucABCD* and *iroN* [7]. In contrast, only a very low percentage of CRKP strains in the United States and the European Union were found to carry these virulence genes. The factors that drive the increasing prevalence and persistence of CR-hvKP in clinical settings in China are not known, which renders it incapable of developing effective intervention strategies to arrest or stop the trend.

Recent studies from China on risk factors for infections and mortality caused by CRKP showed that exposure to antibacterial agents especially carbapenems and tigecycline was one of the risk factors associated with high mortality for CRKP infections [8,9]. Tigecycline was initially approved by the FDA for the treatment of complicated intra-abdominal infections in 2005 [10], followed by approval for treating community-acquired bacterial pneumonia in 2008 [11]. In addition, tigecycline was subjected to clinical trials for the treatment of CRKP infections in China in 2014, and drug combinations of carbapenems and tigecycline have been widely used since then. Despite the in vitro antibacterial activity of tigecycline against many important pathogens, data regarding clinical efficacy have been mixed, with a high mortality risk observed in comparative clinical trials [12]. *Lactobacillus* is regarded as the most well-known probiotic with the favourable properties of biological therapeutics. Many investigations have demonstrated the antimicrobial activity of *Lactobacillus* strains against a wide range of pathogens, including *Staphylococcus aureus* [13], *Salmonella typhimurium* [14], *Escherichia coli* [15], *Pseudomonas aeruginosa* [16] and *K. pneumoniae* [17], in which the proposed antibacterial mechanisms of *Lactobacillus* involve in nutrient and binding sites competition, immune-stimulation, the capability to lower intestinal pH via generation of organic acids such as acetic and lactic acids as well as the production of antimicrobial compounds such as fatty acid and bacteriocins [18,19]. In this study, we created isogenic *K. pneumoniae* strains that harbour a virulence plasmid, a *bla*_KPC-2_-bearing plasmid or both plasmids to simulate the evolution routes by which classic *K. pneumoniae* (cKP) evolves to become CR-hvKP and investigate the effect of tigecycline and meropenem on the intestinal colonization of CR-hvKP and the composition of gut microbiota in a mouse colonization model.

## Materials and methods

### Bacterial strains, plasmids and strain construction

**Figure 1.**
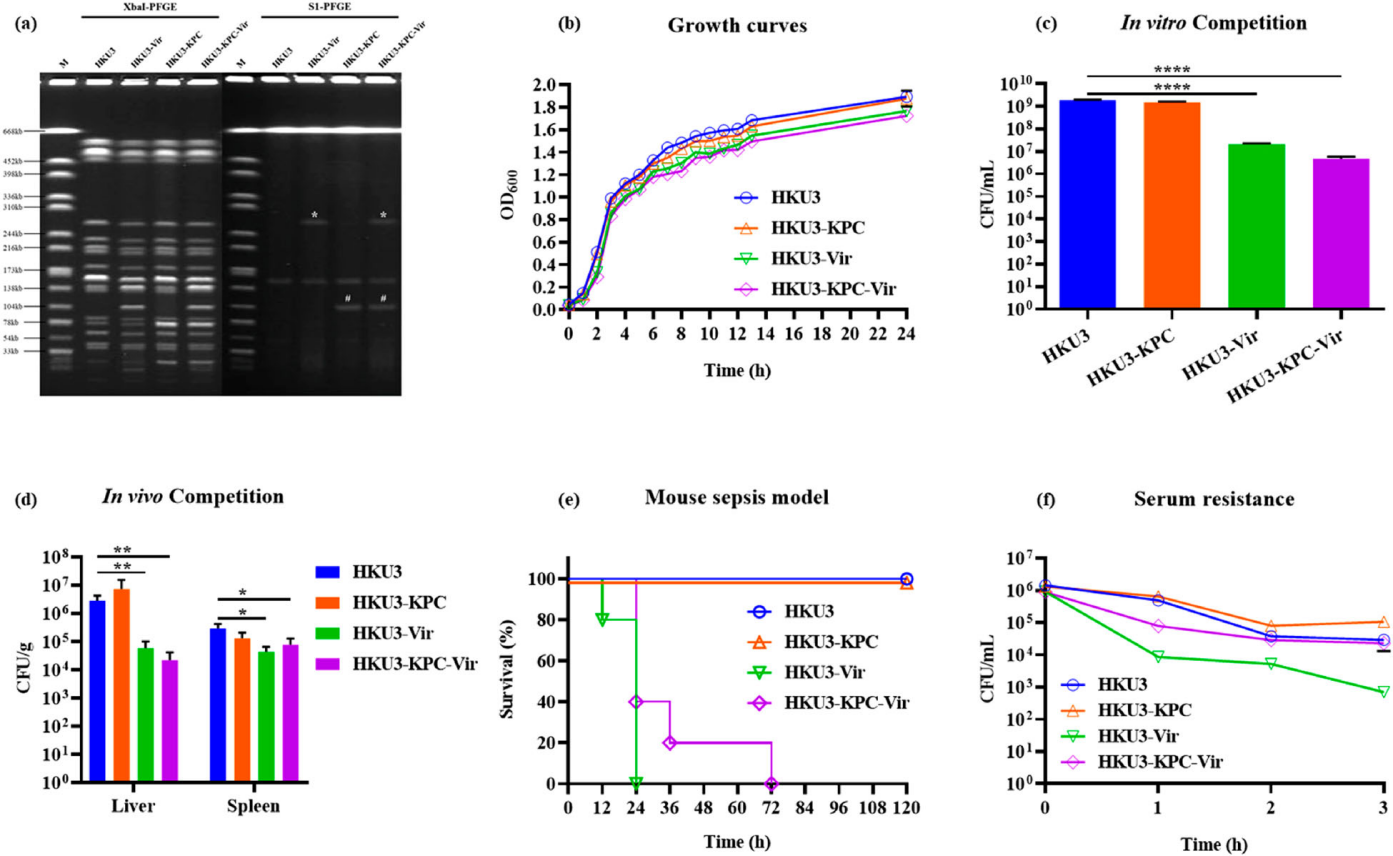
Phenotypic characterization of ST11 *K. pneumoniae* HKU3 and its transconjugants. (a) PFGE profiles of HKU3 and its transconjugants. Asterisk, virulence plasmid; hashtag, *bla*_KPC-2_-bearing plasmid. (b) Growth curves of HKU3 and transconjugants in LB broth. (c) *In vitro* competition in LB broth. (d) *In vivo* competition in mouse sepsis model. Statistical analysis was conducted by unpaired Student’s *t*-test. * *P *< 0.05, ** *P *< 0.01, ****P < 0.0001. (e) Virulence potential of HKU3 and its transconjugants depicted in a mouse sepsis model. Survival of mice infected with 1.0*10^7^ CFU of each *K. pneumoniae* was shown (n = 10). Statistical analysis was performed by Log-rank (Mantel-Cox) test. **** *P *< 0.0001. (f) Serum resistance. Data were presented as mean ± SD of three independent experiments.

Since the ST11 type is the most predominant type of clinical CRKP and CR-hvKP in China, we first created a set of isogenic strains to conduct an unbiased investigation of various genetic and phenotypic features. ST11 *K. pneumoniae* HKU3, a ciprofloxacin-resistant clinical strain which served as a recipient in conjugation experiments, was collected from a local hospital in Hong Kong in 2017. A *bla*_KPC-2_-bearing plasmid p17ZR-91-KPC (MN200129), recovered from a clinical CRKP strain 17ZR-91 [4], was conjugated to HKU3 as previously described [20] to produce the transconjugant HKU3-KPC. A virulence plasmid p16ZR-26-Vir was conjugated to HKU3 and HKU3-KPC to produce transconjugants HKU3-Vir and HKU3-KPC-Vir, respectively. Conjugation experiments were performed as previously described with slight modification [4]. Briefly, transconjugant HKU3-KPC was selected on MacConkey agar supplemented with 0.5 µg/mL meropenem and 32 µg/mL ciprofloxacin. Then, the virulence plasmid was conjugated to HKU3 and HKU3-KPC, respectively. Transconjugant HKU3-Vir was selected on MacConkey agar supplemented with 16 µg/mL potassium tellurite and 32 µg/mL ciprofloxacin. Transconjugant HKU3-KPC-Vir was selected on MacConkey supplemented with 0.5 µg/mL meropenem, 16 µg/mL potassium tellurite and 32 µg/mL ciprofloxacin. Genetic identification of three transconjugants was confirmed by PCR assay [3], minimum inhibitory concentrations (MIC) and pulsed-field gel electrophoresis (PFGE) profiles [21]. Virulence plasmid p16ZR-26-Vir was sequenced and subjected to bioinformatics analysis as previously described [4]. Results showed that plasmid p16ZR-26-Vir was 278,363 bp in length, belonged to IncHI1 type, and harboured various virulence determinants including *rmpA2* and *iucABCDiutA* and genes encoding resistance to tellurium (*terABCD*). PFGE results showed that three transconjugants exhibited identical XbaI-PFGE profiles but different S1-PFGE profiles to the recipient HKU3, suggesting that they were isogenic isolates of HKU3 that contained the virulence and/or *bla*_KPC-2_-bearing plasmids (Figure 1(a)). Briefly, HKU3-KPC was found to contain an extra ∼110 kb plasmid encoding increased MIC to meropenem (Table 1), and HKU3-Vir contained an extra ∼270 kb plasmid encoding increased MIC to tellurite when compared to the recipient HKU3. HKU3-KPC-Vir contained both the ∼110 kb and ∼270 kb plasmids and exhibited increased MIC to both meropenem and tellurite.

**Table 1.** Phenotypic and molecular characterization of ST11 *K. pneumoniae* HKU3 and its transconjugants.

Strain ID	MIC (µg/mL)								*rmp*A2	*bla*_KPC-2_
	MRP	CTX	AMK	CIP	TGC	CT	TE	CAZ		
HKU3	0.06	>128	1	64	1	2	4	16	–	–
HKU3-KPC	64	>128	2	128	1	2	8	64	-	+
HKU3-Vir	0.06	>128	2	64	1	2	>128	8	+	-
HKU3-KPC-Vir	64	>128	1	128	1	1	>128	64	+	+

Note: MRP: meropenem; CTX: cefotaxime; AMK: amikacin; CIP: ciprofloxacin; TGC: tigecycline; CT: colistin; TE: tellurite; CAZ: ceftazidime.

### Growth kinetics

Non-competitive growth assays of *K. pneumoniae* HKU3 and its transconjugants were performed. Overnight culture of test strains was diluted 100-fold in fresh LB broth and grown at 37°C with agitation (250 rpm). Bacterial growth was monitored by measuring the optical density at 600 nm (OD600) until the culture reached the stationary phase. Growth curves were plotted by showing the mean and standard deviation of three independent tests.

### Competition assays and fitness evaluation

To evaluate the impact of plasmid carriage on bacterial fitness, competition assays of HKU3 and its transconjugants were performed both in vitro and in vivo. An in vitro competition experiment was performed as previously described [22]. Four *K. pneumoniae* isolates were collected from exponential cultures and mixed at a ratio of 1:1:1:1 (1.5*10^3^ CFU of each strain). The mixture was then inoculated into 10 mL LB broth and incubated at 37°C for 18 h. The proportion of each strain in the mixture was determined by plating serial 10-fold dilutions on MacConkey agar and MacConkey agar supplemented with 0.5 µg/mL meropenem, 32 µg/mL tellurite, 0.5 µg/mL meropenem and 32 µg/mL tellurite, respectively. The competitive index (CI) was interpreted as the ratio of transconjugant to HKU3. By definition, a CI of 1 suggests that transconjugant has no fitness advantage; a ratio greater than or less than 1 indicates increased or decreased fitness, respectively. An in vivo competition experiment was performed using a mouse infection model. Briefly, ICR mice (weighted 25 g, n = 5) were infected with a mixture of HKU3 and three transconjugants (1.0*10^6^ CFU of each strain) by intravenous inoculation. At 24 h post-infection, mice were sacrificed and organ homogenates including liver and spleen were spread onto MacConkey agar as described above to determine the bacterial load of each transconjugant in different tissues.

### Mouse sepsis model

A mouse sepsis model was used to assess the discrepancy between the virulence level of *K. pneumoniae* HKU3 and three transconjugants. ICR mice (weighted 25 g) were randomly assigned into four groups (n = 10) and intraperitoneally infected with 1.0*10^7^ CFU of HKU3, HKU3-KPC, HKU3-Vir and HKU3-KPC-Vir, respectively. Survival of mice was recorded at 12 h-interval within a 120 h period. Survival curves were delineated using GraphPad Prism 8 and statistical analysis was conducted by the Log-rank (Mantel–Cox) test.

### Serum resistance

Serum resistance was evaluated as previously described [23] in which bacterial suspensions containing 1.5*10^6^ CFU/mL of test strains were obtained from logarithmic cultures, mixed at a ratio of 1:3 (vol/vol) with pooled human serum and incubated at 37°C with agitation. Colony counts were recorded for the initial mixture and after 1, 2 and 3 h of incubation. Serum susceptibility was depicted by measuring bacterial survival rate against incubation time. *K. pneumoniae* was regarded as susceptible to serum if CFU counts dropped to 1% of the initial mixture, and resistant to serum when over 90% of organisms survived after 2 h incubation [24].

### Competitive colonization of *K. pneumoniae* in the rat gastrointestinal tract

Male SD rats (4∼5 weeks, weighted 120 g) were maintained in a standard animal facility. Overnight culture of HKU3 and transconjugants HKU3-KPC, HKU3-Vir and HKU3-KPC-Vir were diluted 100-fold in fresh LB broth and grown at 37°C until the exponential phase was reached. Bacterial cells were harvested and mixed in a ratio of 1:1:1:1 (1.0*10^8^ CFU/mL of each strain). SD rats were fed with 1 mL *K. pneumoniae* mixture using a feeding needle. At 1 h post-infection, rats (n = 4) were assigned into four groups and treated with saline, 10 mg/kg meropenem, 14 mg/kg tigecycline, and both 10 mg/kg meropenem and 14 mg/kg tigecycline, respectively, by intravenous injection. Such treatment was conducted for total 3 times at 24 h-interval. To quantify bacterial shedding, fresh faecal pellets were collected and subjected to serial dilutions and inoculated onto MacConkey agar as described in competition assays. Daily faecal samples collected were stored at −80°C for further faecal microbiome analysis. For the *Lactobacillus* spp. (or acetic acid) feeding experiment, rats were orally infected with a *K. pneumoniae* mixture, followed by treatment with 14 mg/kg tigecycline and feeding with 1.0*10^10^ CFU *Lactobacillus* spp. mixture (*L. reuteri*, *L. murinus*, *L. johnsonii* and *L. intestinalis* isolated from normal rat gut microbiota and mixed in equal proportions) (or 200 µL 50 mM acetic acid) at 1 h post-infection. Tigecycline treatment and *Lactobacillus* spp. (or acetic acid) feeding were conducted for 3 times at 24 h-interval.

### Fecal microbiome analysis

Faecal samples were obtained across five time points, including before treatment (DT-D0), during treatment (DT-D2), after treatment (PT-D3 and PT-D8) and after recovery (PT-D15). Genomic DNA was extracted using the QIAamp Power Fecal Pro DNA Kit (Qiagen). Total DNA was recovered from each sample, yielded sufficient DNA for metagenomic sequencing. DNA samples were subjected to random shotgun sequencing. Briefly, 200 ng DNA was used for metagenomic library preparation. Prepared libraries were quantified, and sequencing was performed on HiSeq X Ten (Illumina) using 2*150 bp read lengths. I7 and I5 indexes were used to process paired forward and reverse reads using the built-in metagenomic workflow as previously described [25]. Thereafter, de novo assembly was performed using SPAdes v3.15.5 [26]. HUMAnN 3 3.0 [27] was used to profile the abundance of mice gut microbiome at the species, genus and phylum levels. Kraken 2 [28] were used to identify the host of antibiotic resistance genes (ARGs) by alignment contigs to the standard database. The Sankey plot was made by Sankey package in R platform. Principal component analysis (PCA) and redundancy analysis (RDA) were performed on the abundance of bacterial species using the vegan package.

### Co-culture assays and killing by acetic acid

The co-culture assay was performed as previously described [29]. *K. pneumoniae* and *Lactobacillus* spp. strains were cultured in their basic broth. Subsequently, *K. pneumoniae* was incubated in modified MRS broth containing overnight *Lactobacillus* spp. suspension obtained by filtering *Lactobacillus* spp. culture using a 0.45 µm membrane. After that, the mixtures were incubated at 37°C. Serial dilutions were plated on LB agar to determine the number of *K. pneumoniae* colonies at each time point. For the killing curve by acetic acid, the *K. pneumoniae* strain was incubated with different concentrations of acetic acid in sterile PBS and incubated at 37°C. The number of *K. pneumoniae* was determined by spreading serial dilutions on LB agar.

### Ethics approval

Animal experiments were approved by the Research Animal Care and Use Committee of the City University of Hong Kong.

## Results

### Phenotypic characteristics of isogenic ST11 *K. pneumoniae* carrying the *bla*_KPC-2_-bearing plasmid and virulence plasmid

Four isogenic strains cKP, CRKP, hvKP and CR-hvKP were generated in this study (Figure 1(a), Table 1). An ST11 *K. pneumoniae* strain HKU3 (cKP), which is the most dominant type of CRKP and CR-hvKP, served as a recipient strain. A *bla*_KPC-2_-bearing plasmid p17ZR-91-KPC (MN200129), recovered from a clinical CRKP strain 17ZR-91 [4], was conjugated to HKU3 as previously described [20] to produce the transconjugant HKU3-KPC (CRKP). A virulence plasmid p16ZR-26-Vir was conjugated to HKU3 and HKU3-KPC to produce transconjugants HKU3-Vir (hvKP) and HKU3-KPC-Vir (CR-hvKP), respectively. The effect of plasmid acquisition on bacterial growth was first assessed in LB broth under a non-competitive environment. Although HKU3 grew slightly faster than three transconjugants, there was no significant difference in growth rates between HKU3 and the plasmid-bearing transconjugants (Figure 1(b)), suggesting that it was a suitable model used in experiments to simulate the process of *K. pneumoniae* infection and investigate the role of different plasmids in mediating selective adaptation of *K. pneumoniae* to the gastrointestinal tract of both animal and human.

### Carriage of virulence and *bla*_KPC-2_-bearing plasmids is associated with decreased fitness

To investigate the effect of plasmid carriage on bacterial fitness, competition assays were performed both in vitro and in vivo (in LB broth and mouse sepsis model, respectively). Results of in vitro competition showed that significantly decreased fitness was observed in both HKU3-Vir and HKU3-KPC-Vir, with a competitive index (CI) being 0.0114 and 0.0025, respectively. It should be noted that HKU3-KPC also exhibited a slightly lower fitness when compared with the recipient HKU3 (Figure 1(c)). These findings suggested that carriage of both *bla*_KPC-2_-bearing plasmid and virulence plasmid conferred a fitness cost on *K. pneumoniae* HKU3, but the virulence plasmid was associated with a higher fitness cost than the *bla*_KPC-2_-bearing plasmid. *In vivo*, the competitive index was determined using a mouse sepsis model, in which systemic dissemination of test strains was allowed to occur. Bacteria recovered from liver and spleen samples of infected mice were used to calculate the CIs of three transconjugants. Data on in vivo competition were similar to that observed in in vitro competition, with both HKU3-Vir and HKU3-KPC-Vir exhibiting a significant reduction in fitness in both livers and spleens (Figure 1(d)); however, a markedly lower fitness level was observed in livers (CI_HKU3-Vir _= 0.0372, CI_HKU3-KPC-Vir _= 0.0090) when compared to strains recovered from spleens (CI_HKU3-Vir _= 0.1780, CI_HKU3-KPC-Vir _= 0.2688). However, HKU3-KPC exhibited a slightly increased fitness in the liver when compared with the recipient HKU3, which was slightly inconsistent with that of in vitro competition, which may be attributed to the effect of immune response imposed in vivo.

### Virulence and serum resistance

The virulence level of HKU3 and three transconjugants was assessed using a mouse infection model. The infection of mice with 1.0*10^7^ CFU HKU3-Vir could cause 100% mortality at 24 h post-infection; such a killing rate was higher than that recorded for HKU3-KPC-Vir, for which 80% mortality at 36 h and 100% mortality at 72 h post-infection were recorded (Figure 1(e)). However, 0% mortality was observed at 120 h post-infection when mice were infected by HKU3-KPC or HKU3. These findings suggest that the acquisition of virulence plasmid significantly enhances the virulence level of ST11 *K. pneumoniae* HKU3, but virulence expression may be slightly impaired by the acquisition of *bla*_KPC-2_-bearing plasmid or the development of carbapenem resistance. Serum resistance is also a feature of virulence exhibited by Gram-negative pathogens that enable bacteria to migrate to distant body sites and cause infections. Therefore, we evaluated the ability of HKU3 and three transconjugants to resist serum-mediated killing. HKU3-Vir was found to be more susceptible to serum and exhibited significantly reduced survival in serum when compared to HKU3, a reduction in survival was also observed in HKU3-KPC-Vir when compared to HKU3-KPC (Figure 1(f)). However, HKU3-KPC-Vir and HKU3-KPC exhibited increased survival when compared to HKU3-Vir and HKU3, respectively. These data indicate that carriage of the virulence plasmid would attenuate the ability of HKU3 to resist serum, yet acquisition of *bla*_KPC-2_-bearing plasmid leads to enhancement in serum resistance in ST11 *K. pneumoniae*.

### Acquisition of virulence plasmid rendered *K. pneumoniae* unable to colonize rat gastrointestinal (GI) tract

**Figure 2.**
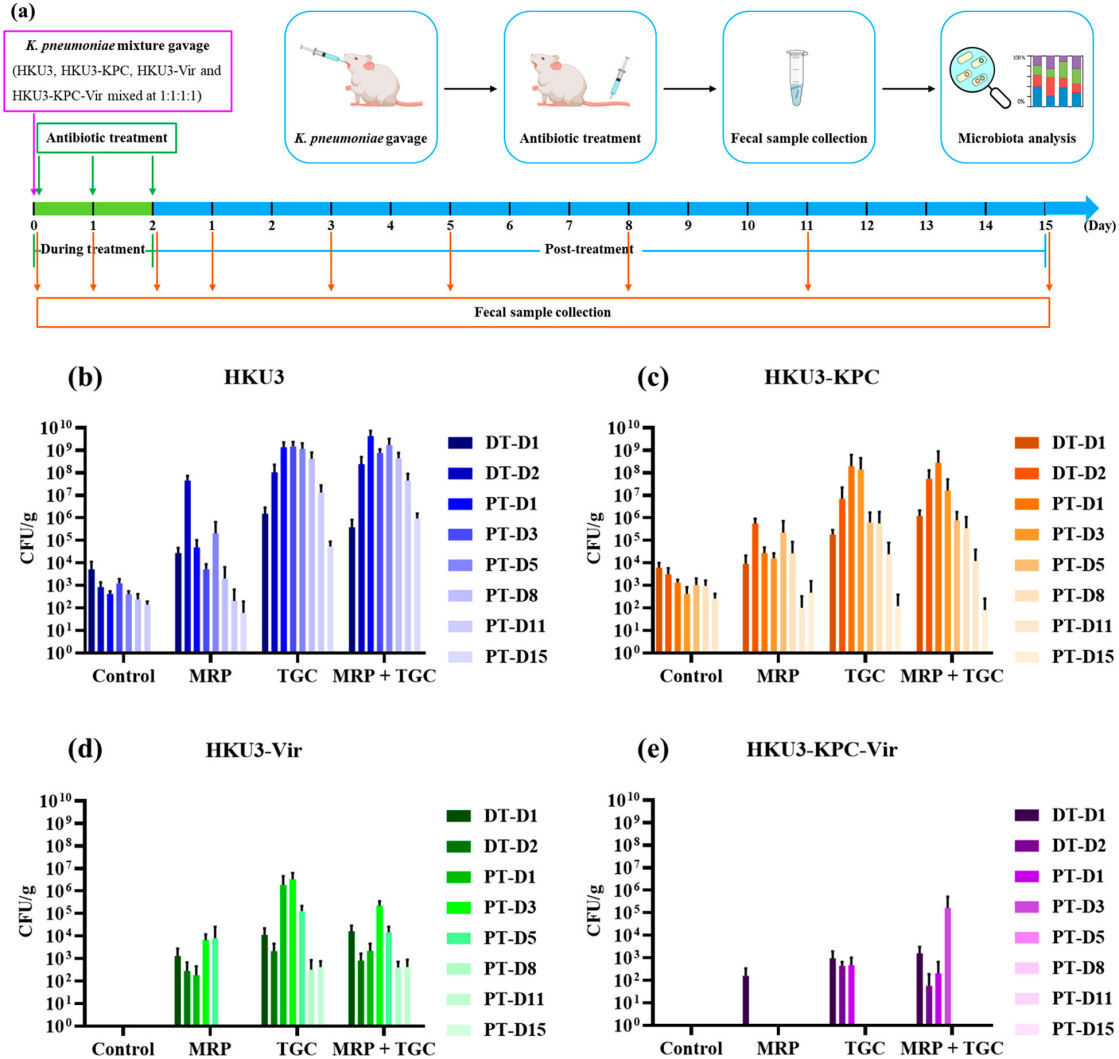
Effect of meropenem and tigecycline on intestinal colonization of *K. pneumoniae* HKU3 and three transconjugants in rats. (a) Experimental design. SD rats were orally infected with a mixture of HKU3 and three transconjugants and subjected to antibiotic treatments (n = 4). Fresh faeces were collected at indicated days for assessment of *K. pneumoniae* shedding and further faecal microbiota analysis. The abundance of *K. pneumoniae* HKU3 (b) and three transconjugants HKU3-KPC (c), HKU3-Vir (d) and HKU3-KPC-Vir (e) colonized in the rat gastrointestinal tract upon different antibiotic treatments was determined during an 18-day experimental period. Data were presented as mean ± standard deviation. DT-D, day during antibiotic treatment; PT-D, day post antibiotic treatment.

Competitive colonization of *K. pneumoniae* HKU3 and its transconjugants was investigated in a rat intestinal colonization model under different antibiotic pressure, in which SD rats were orally infected with a mixture containing an equal amount of four test strains, followed by antibiotic administration (Figure 2(a)). Our data showed that only HKU3 and HKU3-KPC could colonize the rat GI tract at a level of 10^3^–10^4^ CFU/g faeces and persist until PT-D11 in the control group, while HKU3-Vir and HKU3-KPC-Vir were not detectable throughout the experimental period (Figure 2(b–e)). It appears that acquisition of the *bla*_KPC-2_-bearing plasmid by HKU3 did not affect its ability to colonize the rat GI tract; however, the acquisition of the virulence plasmid dramatically impaired the ability of HKU3 to colonize the GI tract. These data may contribute to our understanding of why hvKP strains could not become clinically prevalent even when they have emerged in the 1980s, while CRKP strains have disseminated worldwide.

### Tigecycline facilitated the GI tract colonization of HKU3-Vir and HKU3-KPC-Vir

We then tested the effect of antimicrobial treatment on gut colonization of rats infected with a mixture of HKU3 and three transconjugants. Our data showed that when rats were treated with meropenem, HKU3 and HKU3-KPC could be detected at 10^5^ CFU/g faeces at the beginning and persisted throughout the experimental period, except for a slight decrease in the abundance of HKU3 and HKU3-KPC at the later experimental stage (Figure 2(b,c)). Surprisingly, HKU3-Vir could also be detected in the faeces of meropenem-treated animals; although the level of colonization was low (10^3^–10^4^ CFU/g faeces), it persisted until PT-D5. On the other hand, HKU3-KPC-Vir persisted in the meropenem-treated animal intestinal tract for only one day (Figure 2(d,e)). When rats were treated with tigecycline, however, a high abundance of HKU3 and HKU3-KPC was detectable in the faeces (10^7^–10^9^ CFU/g and 10^6^–10^8^ CFU/g faeces, respectively). Such colonization level was significantly higher than that in the control group. HKU3-Vir was found to colonize the intestinal tract of tigecycline-treated animals with an efficiency of 10^4^–10^6^ CFU/g faeces and persisted until PT-D11. A significantly higher amount and longer persistence was observed for HKU3-Vir when compared to both the control and meropenem-treated groups. HKU3-KPC-Vir in the tigecycline-treated group also exhibited a longer persistence (4 days) than that in the meropenem-treated group, although the number of HKU3-KPC-Vir recorded did not significantly increase. HKU3, HKU3-KPC and HKU3-Vir in rats treated with meropenem and tigecycline combination exhibited a level of colonization similar to those treated with tigecycline alone. However, HKU3-KPC-Vir subjected to treatment with meropenem and tigecycline combination persisted longer than those treated with tigecycline alone (Figure 2(b–e)). These findings indicated that treatment with tigecycline, with or without meropenem, facilitated colonization of not only HKU3 and HKU3-KPC but also HKU3-Vir and HKU3-KPC-Vir. Therefore, we speculate that the clinical use of tigecycline alone or in combination with meropenem results in the persistence of hvKP and CR-hvKP in the human gut microbiome and hence an increased prevalence of CR-hvKP in clinical settings.

### Effect of meropenem and tigecycline on faecal microbiota composition

**Figure 3.**
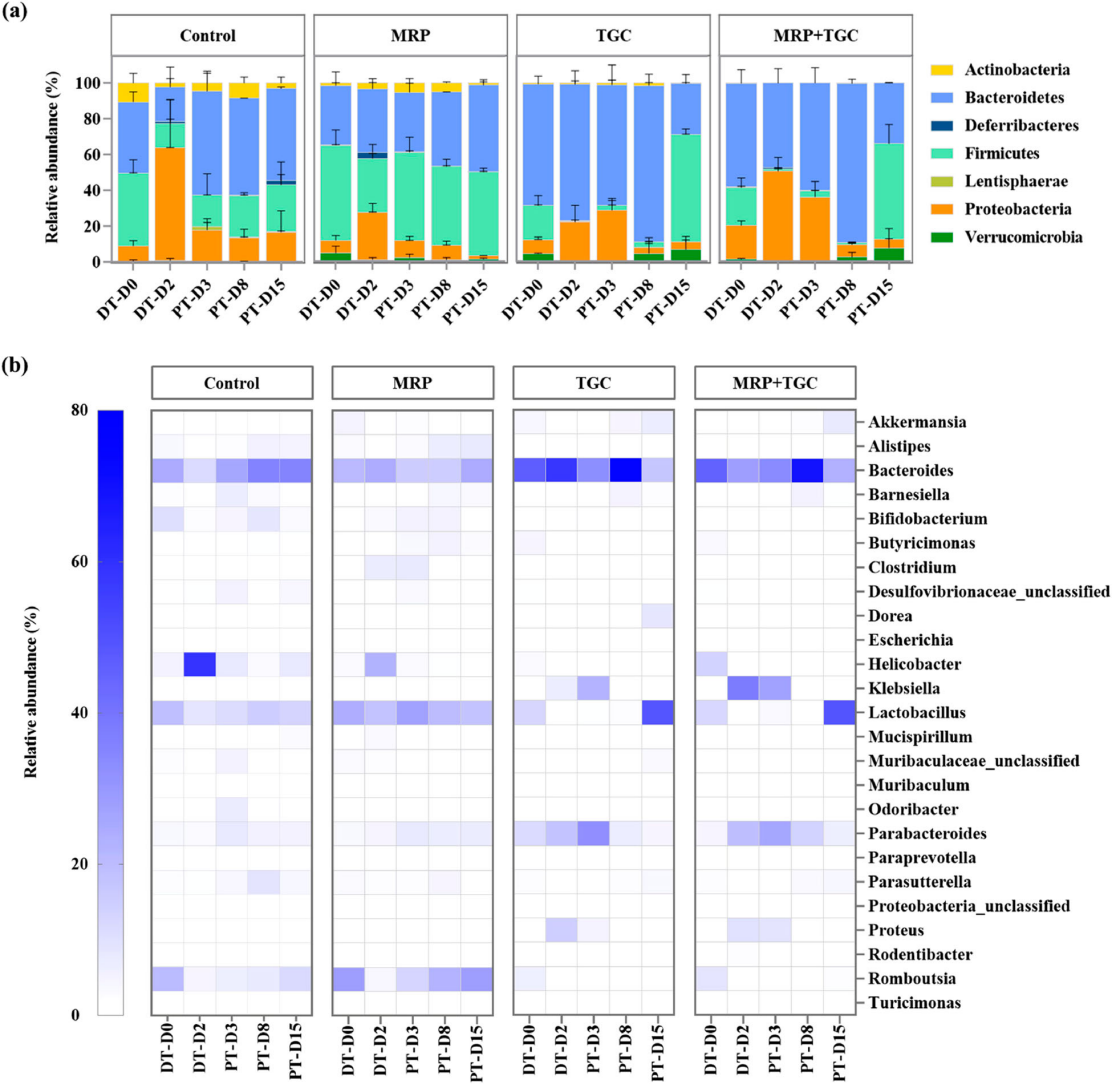
Composition of faecal microbiota. (a) Composition of faecal microbiota at the phylum level. Stacked bar graph showing the relative abundance of dominant organisms (phylum level). The cutoff point for selecting the dominant phyla was set at ≥0.01%. (b) Composition of faecal microbiota at the genus level. Heatmaps depicting the relative abundance of the top 25 bacterial genus detectable. *Y* axis showed the average percentages of sequence reads. DT-D, day during antibiotic treatment; PT-D, day post antibiotic treatment.

Although antibiotics are required to treat bacterial infections, they are also known to disrupt the gut microbiota composition and in turn, create favourable conditions for the overgrowth of opportunistic bacterial pathogens [30–32]. We then analysed the faecal microbiome to explore the effect of meropenem and tigecycline on faecal microbiota composition. Results showed that antibiotic administration caused a reduction in both microbial density and diversity in the GI tract (Figure 3). In a normal rat gut microbiota (DT-D0), *Firmicutes* (∼40%), *Bacteroidetes* (∼40%), *Proteobacteria* (∼10%) and *Actinobacteria* (∼8%) accounted for more than 98% of the total microbiome population (Figure 3(a)). When animals were orally infected with *K. pneumoniae* strains in the control group, the proportion of *Proteobacteria* dramatically increased and *Firmicutes* and *Bacteroidetes* significantly decreased at DT-D2 and quickly recovered to normal levels (Figure 3(a)). A combined analysis of the proportion of *K. pneumoniae* in the microbiota and the number of viable *K. pneumoniae* in plate counting experiments showed that *K. pneumoniae* only constituted a very minor proportion of *Proteobacteria* or total gut microbiota throughout the infection processes. When rats were treated with meropenem, the proportion of *Firmicutes* decreased to ∼30% and *Proteobacteria* increased to ∼27% on DT-D2 and recovered to the normal level quickly (Figure 3(a)). When rats were treated with tigecycline, the population structure of gut microbiota significantly changed on DT-D2, with *Bacteroidetes* (∼76%) becoming the dominant phyla and *Firmicutes* (∼0.6%) almost disappearing (Figure 3(a)). At the genus level, the proportion of *Lactobacillus* and *Romboutsia* in the *Firmicutes* phylum drastically decreased from DT-D2 to PT-D8 and gradually recovered until PT-D15. Consistent with the plate counting data, the proportion of *Klebsiella* (*Proteobacteria*) in the tigecycline treatment group increased to 22.26% (Figure 3(b)). Similar microbiome changes were observed in the combined treatment group. Principal component analysis (PCA) confirmed that *Klebsiella* genera were positively correlated with tigecycline treatment or tigecycline in combination with meropenem during DT-D2 to PT-D3, and control and meropenem treatment were positively correlated with *Lactobacillus* (Fig S1). In addition, antimicrobial resistance gene (ARG) analysis of gut microbiome showed that in control and meropenem groups, *Clostridia* and *Bacilli* were the predominant bacterial host of ARGs with less numbers of genes. In contrast, in the tigecycline group, *Gammaproteobacteria* became the predominant bacterial host of ARGs with large numbers of ARGs including various β-lactamase genes (Fig S2).

### *Lactobacillus* spp*.* reduced the colonization of *K. pneumoniae* induced by tigecycline

**Figure 4.**
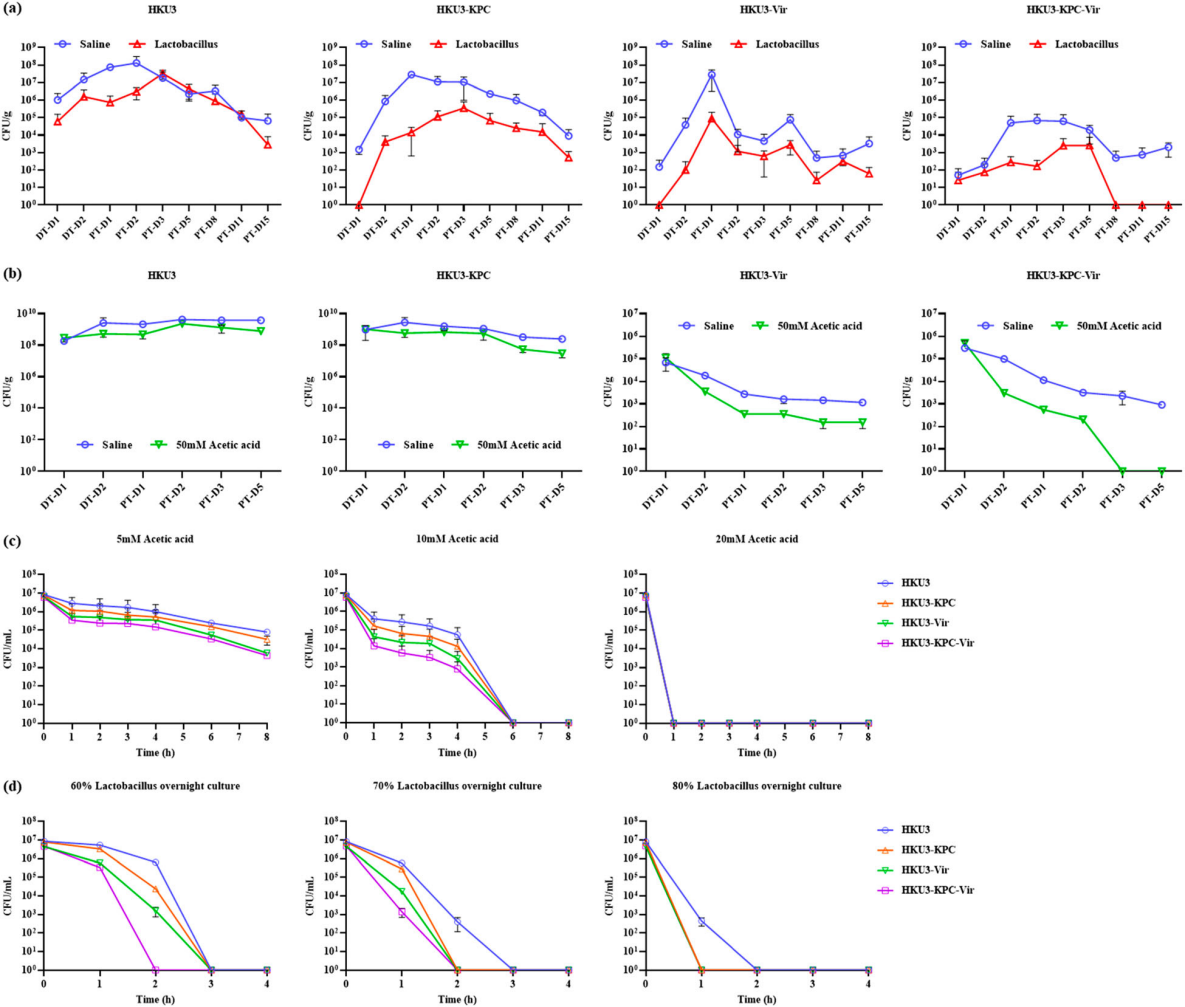
Effect of *Lactobacillus* spp. and acetic acid on intestinal colonization of *K. pneumoniae* HKU3 and its transconjugants. Abundance of *K. pneumoniae* HKU3 and three transconjugants HKU3-KPC, HKU3-Vir and HKU3-KPC-Vir colonized in GI tract upon feeding with *Lactobacillus* spp. (a) and acetic acid (b). *P *< 0.0001. DT-D, day during antibiotic treatment; PT-D, day post-antibiotic treatment. Killing curves of *K. pneumoniae* HKU3 and three transconjugants in acetic acid (c) and *Lactobacillus* spp. overnight culture (d).

Based on the above analysis, we speculated that the proportion of *Lactobacillus* in gut microbiota may be associated with *K. pneumoniae* colonization. To verify our hypothesis, *Lactobacillus* spp*.* feeding experiment was conducted on rats orally infected with *K. pneumoniae* and subjected to tigecycline treatment. Upon *Lactobacillus* spp*.* feeding, intestinal colonization of *K. pneumoniae* significantly decreased in tigecycline-treated rats (Figure 4(a)). Specifically, the amount of HKU3 in *Lactobacillus* spp. feeding group decreased by one to two orders of magnitude during DT-D1 to PT-D2 with the largest reduction observed on PT-D1 when compared to that in the saline group. Upon *Lactobacillus* spp. feeding, the amount of HKU3-KPC and HKU3-Vir decreased by two to three orders of magnitude throughout the experiment, with the first detected time postponing to DT-D2. In addition, the amount of HKU3-KPC-Vir also significantly decreased in which it was even undetectable after PT-D8 (Figure 4(a)). These findings suggested that *Lactobacillus* spp. feeding could reduce the colonization of *K. pneumoniae* induced by tigecycline treatment, especially significantly reducing the colonization of CR-hvKP in both the colonization quantity and duration.

### *In vivo* anticolonization and in vitro antibacterial activity of acetic acid against CR-hvKP strain

Investigations have illustrated that the antimicrobial activities of *Lactobacillus* on pathogenic *E. coli*, *Salmonella* and *Campylobacter jejuni* were attributed to the organic acids they produced [33]. It has also been demonstrated that acetic and lactic acids were the major organic acids involved in the antibacterial effects of *Lactobacillus* strains [17]. Recent studies have reported that the anticolonization of *K. pneumoniae* by *Lactobacillus* spp. was attributed to increased acetic acid and regulation in microbiota in mice intestinal [29,34]. To investigate the mechanism underlying the inhibition of CR-hvKP colonization by *Lactobacillus* spp., we repeated the *K. pneumoniae* colonization experiment and replaced *Lactobacillus* spp. with acetic acid. As shown in Figure 4(b), 50 mM acetic acid significantly reduced the colonization of HKU3-Vir and HKU3-KPC-Vir, in which the anticolonization capacity was comparable to *Lactobacillus* spp*.* However, the anticolonization ability of acetic acid on HKU3 and HKU3-KPC was inferior to *Lactobacillus* spp. (Figure 4(b)), which might be attributed to the regulation of *Lactobacillus* spp. on the intestinal microbiota. In addition, the inhibition effect of acetic acid and *Lactobacillus* spp. overnight culture on *K. pneumoniae* was also evaluated in vitro. We found that 10 mM acetic acid could effectively inhibit HKU3 and its transconjugants in vitro, and 20 mM acetic acid completely eradicated HKU3 and three transconjugants in the initial stage (Figure 4(c)). We further tested the killing effect of *Lactobacillus* spp. overnight culture on *K. pneumoniae* strains with results showing that it exhibited more effective killing of HKU3-Vir and HKU3-KPC-Vir than HKU3 and HKU3-KPC at concentrations of 60% and 70% (Figure 4(d)). Taken together, these findings suggested that *Lactobacillus* spp. inhibited the colonization of hvKP and CR-hvKP probably due to the accumulation of acetic acid in the intestinal tract.

## Discussion

In recent years, various important *K. pneumoniae* strains have evolved including CRKP, hvKP and CR-hvKP. Plasmids that carry genes encoding virulence and carbapenem resistance play an important role in the evolution of CR-hvKP. However, successful transmission of virulence and resistance plasmids mainly depends on the fitness cost they impose on the hosts [35], which often manifests as decreased growth rate and attenuated competitive ability. Although fitness cost could be alleviated by compensatory evolution [36], the initial cost imposed by plasmid carriage is the principal mechanism of restriction of plasmid replication. There were a few reports on the fitness cost and virulence of carbapenemase production in *K. pneumoniae*. Lavigne et al. reported that the presence of the *bla*_KPC-2_ gene resulted in a reduction of the virulence of *K. pneumoniae* in *Caenorhabditis elegans* [37]. Our previous study reported that a hybrid virulence plasmid expressed increased virulence and different levels of fitness cost in different *K. pneumoniae* strains [38]. However, Jin et al. reported a hybrid plasmid co-harbouring *bla*_KPC-2_ and virulence genes with enhanced virulence in clinical ST11 CRKP, but plasmid stability could be maintained without any observable fitness cost [39]. Findings in this work showed that the *bla*_KPC-2_-bearing plasmid was not associated with a fitness cost or phenotypic virulence in HKU3, while the virulence plasmid conferred significantly enhanced virulence but impaired fitness on HKU3. These data may explain why even though hvKP strains emerged in the 1980s, their prevalence in clinical settings is still very low.

This study demonstrated that meropenem and tigecycline significantly prolonged the period during which HKU3-Vir and HKU3-KPC-Vir colonized the GI tract, which was primarily due to the disruption of normal gut microbiota after treatment with tigecycline and meropenem, resulting in a dramatic increase in the proportion of *Proteobacteria* and a decrease in the proportion of both *Bacteroidetes* and *Firmicutes* in gut microbiota. This finding was consistent with a previous study that tigecycline treatment resulted in major alteration and low diversity in gut microbiota, including a significantly reduced proportion of *Bacteroidetes* and increased prevalence of *Proteobacteria* [40]. It was suggested that an increased prevalence of *Proteobacteria* in the microbial community might be a signature of dysbiosis and a potential diagnostic criterion for various diseases [41]. Consistently, we also demonstrated that tigecycline led to increased numbers and types of important ARGs in the gut microbiome, which might facilitate the further spread of multidrug-resistant (MDR) pathogens in hospitals. Our data exhibited that the most dramatic disruption of the microbiome is attributed to the dramatic reduction of *Lactobacillus* spp., which has been largely recognized as probiotics contributing to human health and blockage of pathogen colonization in the human gut [17,42]. Previous studies have demonstrated that *Lactobacillus plantarum* LP1812 exhibited significant CRKP anticolonization ability through regulating the microbiota and accumulating acetic acid intestinal tract in mice [29], which was consistent with the findings in this study that *Lactobacillus* spp. feeding dramatically reduced the colonization of *K. pneumoniae* HKU3 and three transconjugants in particular HKU3-KPC-Vir in the GI tract. Studies have reported that acetic acid in *Lactobacillus* spp. the supernatant was an important factor in inhibiting *K. pneumoniae* growth and further illustrated that acetic acid inhibited *K. pneumoniae* strains in vitro by acidifying their intracellular environment and subsequently damaging bacterial vigour and fecundity [17,29]. Our study demonstrated the ability of acetic acid to suppress CR-hvKP growth both in vivo and in vitro. The better colonization of hvKP and CR-hvKP in the GI tract of patients may undergo faster person-to-person transmission, especially in a hospital environment [43]. In addition, gut colonization by MDR bacteria has been regarded as a risk factor for the development of infections of high mortality [44]. Taken together, our data suggested that *Lactobacillus* spp. may become a potential candidate for anticolonization strategies against CR-hvKP.

FDA recently warned of higher mortality among patients treated with tigecycline when compared to treatment with other drugs [45]. Previous studies also reported an increased risk of death in patients treated with tigecycline, especially among patients suffering ventilator-associated pneumonia [46,47]. However, due to the lack of choices of effective treatment for clinical CRKP, tigecycline has been approved to be used alone or in combination with carbapenems to treat infections caused by CRKP since 2014 [48]. Clinical use of tigecycline aligns well with the increasing trend of CR-hvKP in clinical settings in China. Several epidemiological studies have demonstrated the increasing prevalence of CR-hvKP in particular ST11 type in China [6,49,50]. In addition, tigecycline is less commonly used in other countries in the world and the prevalence of CR-hvKP is very low outside China. Data from this study provided supportive evidence to confirm the association between tigecycline usage and the prevalence of CR-hvKP in China. We speculated that stopping or reduced the use of tigecycline should impede the persistence and spread of CR-hvKP in clinical settings in China.

This study constructed isogenic *K. pneumoniae* strains to simulate the evolution of cKP to CRKP, hvKP and CR-hvKP and identify factors that facilitate the dissemination of CR-hvKP in clinical settings. Acquisition of virulence plasmid would attenuate the ability of *K. pneumoniae* to colonize the GI tract; however, the usage of tigecycline would facilitate the colonization of CR-hvKP in the animal gut. Notably, we demonstrated that *Lactobacillus* spp. was a potential candidate for anticolonization strategy against CR-hvKP. The findings in this study provided important insights into factors that facilitate the dissemination of CR-hvKP and potential approaches to arresting further dissemination of CR-hvKP. It is also necessary to develop new therapeutic guidelines to treat CR-hvKP infections and establish an extensive collaborative network of scientific and medical personnel to tackle this daunting task.

## Supplementary Material

Supplementary_materials

## Data Availability

The complete sequence of virulence plasmid p16ZR-26-Vir has been deposited into the GenBank database under the accession number OP168824. Metagenomic sequencing data has been deposited into the NCBI database under the BioProject accession number PRJNA909428.
