# Supplementary material for: Clinical use of tigecycline may contribute to the widespread dissemination of carbapenem-resistant hypervirulent Klebsiella pneumoniae strains
Source: Emerg Microbes Infect. 2024 Jan 19;13(1):2306957. doi: 10.1080/22221751.2024.2306957 (PMC10829843; doi:10.1080/22221751.2024.2306957)
Supplement: Supplementary_materials [file TEMI_A_2306957_SM9691.docx]

Supplementary materials

**Clinical use of tigecycline may contribute to the widespread dissemination of carbapenem-resistant hypervirulent *Klebsiella pneumoniae* strains**

Miaomiao Xie^1,2#^, Lianwei Ye^1#^, Kaichao Chen^2#^, Qi Xu^1^, Chen Yang^1^, Xiangnan Chen^1^, Edward Wai-Chi Chan^3^, Fuyong Li^1^, Sheng Chen^2*^

1 Department of Infectious Diseases and Public Health, Jockey Club College of Veterinary Medicine and Life Sciences, City University of Hong Kong, Kowloon, Hong Kong.

2 Department of Food Science and Nutrition, Faculty of Science, The Hong Kong Polytechnic University, Kowloon, Hong Kong.

3 State Key Lab of Chemical Biology and Drug Discovery, Department of Applied Biology and Chemical Technology, The Hong Kong Polytechnic University, Kowloon, Hong Kong.

# These authors contributed equally.

Corresponding author: Sheng Chen, E-mail: sheng.chen@polyu.edu.hk, The Hong Kong Polytechnic University, Kowloon, Hong Kong.

**Keywords**: Carbapenem-resistant hypervirulent *Klebsiella pneumoniae*, tigecycline, virulence plasmid, fitness, colonization

**Supplementary figures**


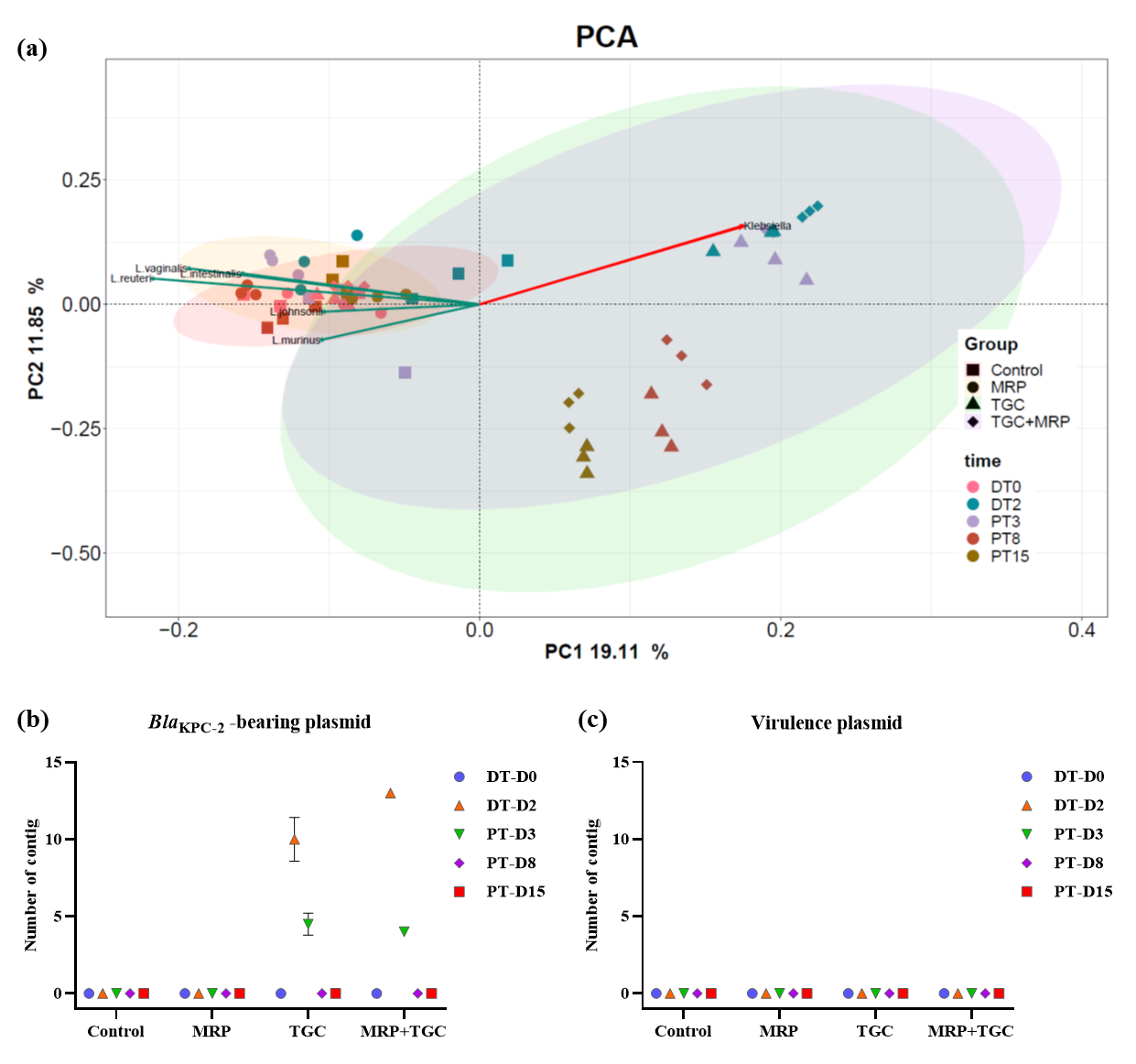


**Supplementary Figure S1. Principal component analysis.** A principal component analysis was performed to visualize the differences of bacterial species in microbiota upon different treatments within a timeframe of 18 days which covered the period before, during and after antibiotic treatment.


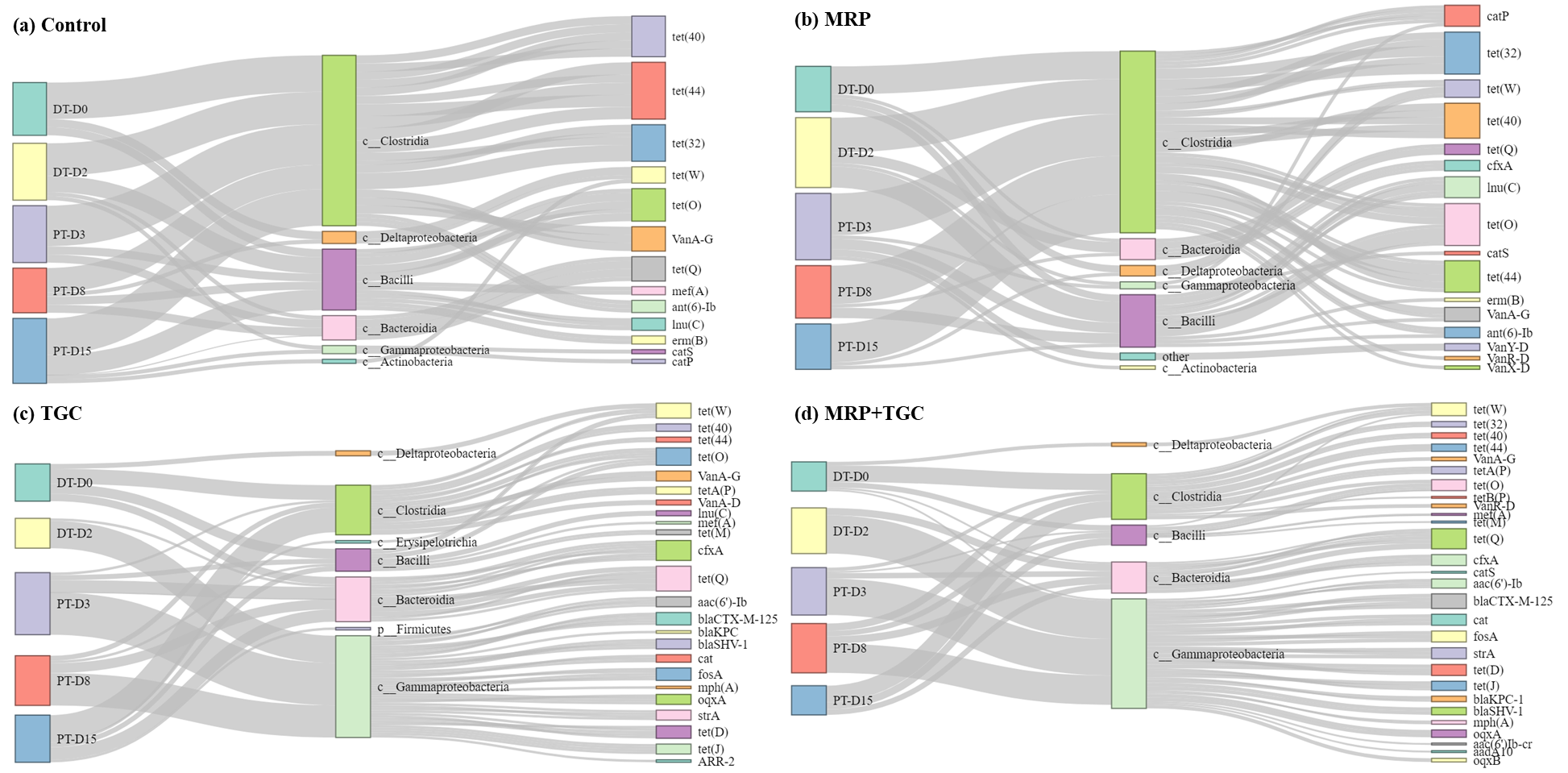


**Supplementary Figure S2. Abundance and hosts of antibiotic resistance genes in rat gastrointestinal microbiota.** Sankey diagram depicting the distribution of antibiotic resistance genes among different bacterial classes upon treatment of saline (**a**), meropenem (**b**), tigecycline (**c**) and meropenem in combination with tigecycline (**d**) within a timeframe of 18 days which covered the period before, during and after antibiotic treatment. The flow bars (gray) indicated the abundance of resistance genes.
